# The Prevalence and Genetic Diversity of Porcine Circoviruses (PCVs) during 2017–2023 in Guangdong Province, China

**DOI:** 10.3390/ani13233640

**Published:** 2023-11-24

**Authors:** Wenke Lv, Lihua Cao, Lulu Yang, Nina Wang, Zhili Li, Shujian Huang, Feng Wen, Jinyue Guo

**Affiliations:** College of Life Science and Engineering, Foshan University, Foshan 528231, China; lvwenke1026@163.com (W.L.); clh270042@163.com (L.C.); 13425999180@163.com (L.Y.); wang_nina@163.com (N.W.); pinganzhili@163.com (Z.L.); huangshujian@fosu.edu.cn (S.H.); wenf@fosu.edu.cn (F.W.)

**Keywords:** pigs, PCV2, PCV3, prevalence, phylo-genetic analysis, genotype

## Abstract

**Simple Summary:**

Porcine circovirus (PCV2 and PCV3) currently stands as the primary viral agent afflicting intensive pig farming, inflicting substantial economic losses on farms. This study endeavored to explore the prevalence and genetic diversity of PCVs that have circulated in Guangdong province in recent years, a prominent hub for pig production within China. In the current investigation, the prevalence of PCV2, PCV3, and PCV2/3 co-infection was found to be 56.48%, 8.81%, and 8.81%, respectively. Nonetheless, no evidence of PCV4 presence materialized in the course of this study. Moreover, the co-occurrence of PCV2 with classical swine fever virus (CSFV), porcine reproductive and respiratory syndrome virus (PRRSV), pseudo-rabies virus (PRV), and porcine epidemic diarrhea virus (PEDV) emerged as a common phenomenon, with the co-infection rate with PEDV (23.85%) surpassing that of PRV (9.17%), PRRSV (18.35%), or CSFV (0.92%). Subsequently, a detailed analysis of the Cap protein sequence of the PCV2 and PCV3 isolates unveiled several high-frequency mutation sites within the epitope regions. Collectively, this study imparts valuable insights into the prevalence and evolution of PCV2 and PCV3 in China between 2017 and 2023, thereby contributing to the formulation of efficacious prevention and control strategies.

**Abstract:**

Porcine circovirus disease poses a significant threat to the pig farming industry. Globally, four genotypes of porcine circovirus are circulating, with porcine circovirus type 2 and 3 (PCV2 and PCV3) being most strongly associated with clinical manifestations. The recently discovered porcine circovirus type 4 (PCV4) exhibits clinical symptoms resembling porcine dermatitis nephropathy syndrome. This study aimed to assess the prevalence and genetic characteristics of PCVs in Guangdong province, China. A comprehensive analysis was conducted on 193 samples collected from 83 distinct pig farms during the period of 2017–2023. A conventional PCR was employed to investigate the presence of PCV2, PCV3, and PCV4. Among the samples, 56.48%, 8.81%, and 8.81% tested positive for PCV2, PCV3, and PCV2/3 co-infection, respectively. Interestingly, PCV4 was not detected. Whole-genome sequencing was performed on 80 PCV2 isolates and 7 PCV3 isolates. A phylo-genetic analysis revealed that 12 strains belonged to PCV2a, 8 strains belonged to PCV2b, and 60 strains belonged to PCV2d, indicating the prevailing presence of PCV2d in Guangdong province, China. Furthermore, two PCV3 isolates were classified as PCV3a and five strains as PCV3b. Notably, an in-depth analysis of the Cap protein sequence of the PCV2 and PCV3 isolates identified high-frequency mutation sites located in predicted epitope regions. Overall, this study provides valuable insights into the prevalence and evolution of PCV2 and PCV3 during the period of 2017–2023 in Guangdong province, China, thereby contributing to the development of effective prevention and control measures.

## 1. Introduction

Porcine circoviruses (PCVs) are a class of single-stranded negative-sense circular DNA viruses belonging to the Circoviridae family and the Circovirus genus [1,2]. The PCV group comprises PCV1, PCV2, PCV3, and, most recently, PCV4. PCV1 was initially detected as a contaminant of PK-15 cell cultures in 1974, and was not associated with a disease [2]. Conversely, PCV2 is considered the primary causative agent of porcine-circovirus-associated disease (PCVAD), which elicits diverse clinical symptoms, such as post-weaning multi-systemic wasting syndrome (PMWS), porcine dermatitis and nephropathy syndrome (PDNS), porcine respiratory disease complex (PRDC), congenital tremors (CT), reproductive failure, and enteric disease in pigs [3,4]. Notably, PCV3 was first characterized in the United States in 2015, and was associated with distinct symptoms, including reproductive disorders, PDNS, respiratory failure, and multi-systemic and cardiac inflammation [5,6]. Lastly, PCV4, initially identified in Hunan province, China, in 2019, has been reported in recent years in Henan, Guangxi, Sichuan, Shaanxi, Shanxi provinces, and Inner Mongolia in China [7,8,9,10,11,12]. It has been suspected to be associated with severe clinical conditions, such as PDNS, respiratory signs, and enteric manifestations [7,8,11,13].

PCV2 is a ubiquitous pathogen with a global footprint [14,15]. Its genome spans 1766–1769 nucleotides (nt) and encompasses two prominent open reading frames (ORFs) [16]. ORF1 encodes two replication-related proteins, Rep and Rep’ [17], while ORF2 encodes the capsid protein (Cap), which serves as the distinctive structural protein and primary antigenic determinant of PCV2 [18]. The sequences within ORF2 exhibit considerable variability and are frequently employed in phylo-genetic analyses of PCV2. Presently, PCV2 is categorized into eight genotypes (PCV2a–PCV2h) in accordance with the widely accepted genotyping methodology [19]. Furthermore, a novel genotype, PCV2i, has been identified in the United States [20]. Since its initial identification, PCV2 has undergone two transitions in its epidemic genotype, from PCV2a to PCV2b in approximately 2003, and, subsequently, from PCV2b to PCV2d in approximately 2012 [21].

Since its discovery in the United States, PCV3 has spread to countries such as Korea, China, Thailand, Japan, Brazil, Italy, Spain, Russia, Germany, and others, indicating its worldwide distribution and significant economic impact on the swine industry [22,23,24,25,26,27,28,29,30,31]. PCV3′s length varies within the range of 1999–2001 nt [5,6]. Similar to PCV2, PCV3 also possesses two primary ORFs (ORF1 and ORF2), which encode the Rep and Cap proteins, respectively. Based on the complete coding sequence of PCV3, it can be classified into two clades: PCV3a and PCV3b [32]. Notably, the evolution rate of PCV3 has been found to be much slower (10^−5^–10^−6^ substitution/site/year) than that of PCV2 [33].

PCVs, classical swine fever virus (CSFV), porcine reproductive and respiratory syndrome virus (PRRSV), pseudo-rabies virus (PRV), and porcine epidemic diarrhea virus (PEDV) are the primary pathogens in pigs that cause huge economic losses to the swine industry [34,35,36,37]. Previous studies have shown that the co-infection of PCV2 with other pathogens can exacerbate the severity of clinical signs, complicate the infection status, and pose challenges to disease prevention and control efforts [35,36,38,39,40]. Therefore, it is imperative to monitor the prevalence of PCVs in order to gain a comprehensive understanding of their impact on pig health and to develop effective strategies to mitigate their adverse effects. Although there have been several reports on the prevalence and genetic variation of PCV2 and PCV3 in some districts of China [41,42,43,44,45,46,47], the information about Guangdong province has been limited in recent years. The aim of this study was to assess the prevalence and genetic characteristics of PCVs in Guangdong province, China.

## 2. Materials and Methods

### 2.1. Sample Collection

**Figure 1:**
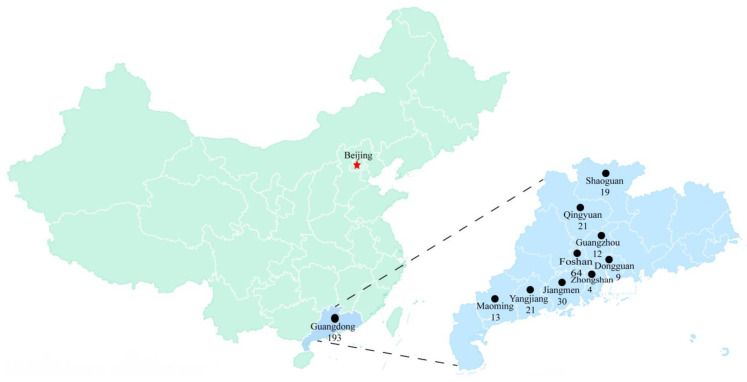
The geographical distribution of 193 porcine clinical samples were marked with black dots in Guangdong province of China. The numbers listed in the figure indicated the total number of samples collected in 9 cities within Guangdong province during 2017–2023.

A total of 193 clinical specimens (including cerebral tissue, pulmonary tissue, lymph nodes, intestinal tissue, splenic tissue, hepatic tissue, and serum) (Table S1) were collected from pigs of different age groups across 83 distinct pig farms in 9 cities within Guangdong province during 2017–2023 (Figure 1). In addition, among the tested 83 swine farms, 42 swine farms received the PCV2 vaccines. The clinical presentations encompassed respiratory symptoms, neurological symptoms, sow abortions, stillbirths, and diarrhea. Standardized operating procedures were followed during sample processing.

### 2.2. Viral DNA Extraction and Virus Detection

The tissue samples, weighing approximately 200 mg, were dissected into smaller fragments and diluted with 5-fold phosphate-buffered saline (PBS). Subsequently, the samples underwent grinding using a grinder, followed by two cycles of a freeze–thaw treatment to facilitate the release of viral particles. Afterward, the samples were subjected to centrifugation at 12,000× *g* for 5 min at 4 °C. The resulting supernatants, measuring 200 μL, were carefully collected for the subsequent viral DNA extraction. This extraction was performed using the Axygen^®^ body fluid viral DNA/RNA Miniprep Kit (Corning, CA, USA), strictly following the manufacturer’s recommended protocol. PCV2, PCV3, and PCV4 were detected via a conventional PCR. The primers used for the detection of PCV2, PCV3, and PCV4 are enumerated in Table 1 [6,8,42,48,49,50]. Briefly, the reactions were conducted in a total volume of 25 μL, containing 12.5 μL of 2 × Taq Plus Master Mix Ⅱ (Dye Plus) (Vazyme, Nanjing, China), 1 μL of each primer (10 μM), 2 μL of extracted DNA, and 9.5 μL of ddH_2_O. The PCR amplification conditions were as follows: initial denaturation at 95 °C for 5 min, followed by 35 cycles consisting of denaturation at 95 °C for 15 s, annealing at 55 °C for 20 s, extension at 72 °C for 30 s, and with a final extension at 72 °C for 10 min. The presence of the amplified PCR products for PCV2, PCV3, and PCV4 was determined by detecting bands at 500, 357, and 391 bp, respectively, on 2% agarose gel.

### 2.3. Detection of Other Pathogens

In addition to identifying PCV2, PCV3, and PCV4, various pathogens, including PEDV, CSFV, PRV, and PRRSV, were discerned by virtue of the distinctive clinical manifestations observed in porcine subjects. Viral DNA/RNA was extracted using the aforementioned Axygen^®^ body fluid viral DNA/RNA Miniprep Kit. Except for PRV, all the other pathogens were RNA viruses, thus, cDNA was synthesized using a reverse transcription reagent (Takara, Dalian, China). The cDNA synthesis reaction system consisted of 5.5 μL RNase-free H_2_O, 2.5 μL gDNA eraser buffer, 1 μL gDNA eraser, and 1.5 μL total RNA. The reaction mixtures described above were incubated at 42 °C for 2 min. Subsequently, 1 μL Primescript RT Enzyme Mix, 1 μL RT Primer mix, 4 μL 5 × Primescript buffer, and 4 μL RNase-free H_2_O were added at 37 °C for 15 min, followed by incubation at 85 °C for 5 s, and, finally, stored at −80 °C until use. The primers used for the detection of PEDV, CSFV, PRV, and PRRSV are listed in Table 1. The PCR reaction mixtures and amplification conditions were as previously described.

### 2.4. Complete-Genome Sequencing of PCV2 and PCV3

In order to obtain the entire genomic sequences of PCV2 and PCV3 for the subsequent phylo-genetic analysis, we used the primers listed in Table 1. The PCR was performed in 50 μL reaction mixtures containing 25 μL TransTaq^®^ DNA Polymerase High-Fidelity (HiFi) (TransGen, Beijing, China), 1 μL of each primer pair (10 μM), 4 μL of DNA template from positive samples, and 19 μL of ddH_2_O. The PCR amplification conditions were as follows: initial denaturation at 94 °C for 5 min, followed by 35 cycles comprising denaturation at 94 °C for 15 s, annealing at 57 °C for 20 s, extension at 72 °C for 90 s, and a final extension at 72 °C for 10 min. The amplified PCR products were analyzed using 1% agarose gel. The desired bands were purified using the Gel Extraction Kit (Accrute Biology, Changsha, China), according to the manufacturer’s protocol, and, subsequently, cloned into the pMD18-T Vector (Takara, Dalian, China). The ligated products were introduced into competent Escherichia coli Trans5α cells (TransGen, Beijing, China) through a transformation. Positive clones were identified via the PCR and sequenced in a commercial facility (Sangon Biotech, Shanghai, China).

### 2.5. Alignment and Phylo-Genetic Analysis

The reference strains and genomic sequences of the PCV2 and PCV3 strains obtained in this study are summarized in Supplementary Tables S2–S4. The complete coding sequences (ORF1 + ORF2) of PCV3 were analyzed and divided into two sequences, ORF1 (Rep) and ORF2 (Cap), with the non-coding regions excluded. Considering that ORF2 was oriented in the opposite direction, it was reversed and then concatenated with ORF1. The PCV3 sub-types were proposed by Li et al. [32].

In this study, the complete coding sequences of PCV2 and PCV3, as well as the ORF2 sequences of the PCV2 strains, were aligned using Clustal W of the Molecular Evolutionary Genetics Analysis program, version 7.0 (MEGA 7.0). Phylo-genetic trees were generated using the maximum likelihood (ML) method in MEGA 7.0, employing a p-distance model and 1000 bootstrap replicates. The amino acid homology was also analyzed using DNAStar (Version 7.0) with the Clustal W method, based on the amino acid sequences of ORF1 and ORF2 of PCV2 and ORF2 of PCV3, respectively.

## 3. Results

### 3.1. Prevalence of PCVs in Guangdong Province, China

To evaluate the prevalence of PCV2, PCV3, and PCV4 in pigs, a comprehensive investigation was conducted in Guangdong province. DNA was extracted from 193 samples and, subsequently, analyzed using specific primers through a conventional PCR. The positive rates of PCV2 were found to be 56.48% (109 out of 193 clinical samples) and 67.47% (56 out of 83 farms). Similarly, the positive rates of PCV3 were 8.81% (17 out of 193 clinical samples) and 12.05% (10 out of 83 farms). Interestingly, the co-infection rate of PCV2 and PCV3 was 8.81% (17 out of 193 clinical samples) and 12.05% (10 out of 83 farms), respectively. However, PCV4 was not detected in any of the clinical samples (Table 2).

In our study, we successfully acquired a total of 80 complete genomes of PCV2 and 7 complete genomes of PCV3, which were deposited in the NCBI GenBank. The corresponding accession numbers are provided in Table S2.

### 3.2. Co-Infection with Other Porcine Viruses

In addition to the co-infection of PCV2 and PCV3, we also explored the co-infection rates of these PCVs with CSFV, PRRSV, PRV, and PEDV. Among the PCV2-positive samples, co-infection with PEDV, PRRSV, and PRV was observed in 23.85% (26/109), 18.35% (20/109), and 9.17% (10/109) of cases, respectively. Only one case of PCV2 co-infection with CSFV was identified. Notably, the co-infection rate of PCV2 with PEDV was higher compared to the other three viruses. Moreover, triple or quadruple infections involving these viruses were also detected (Table 3). Overall, 55.05% (60 out of 109) of the PCV2-positive samples were co-infected with at least one other virus. These findings, in accordance with previous studies, supported the notion that these four viruses are prevalent in Chinese pig herds, and that the co-infection of PCV2 with other viruses is a common occurrence.

### 3.3. Genetic Analysis of PCV2

**Figure 2:**
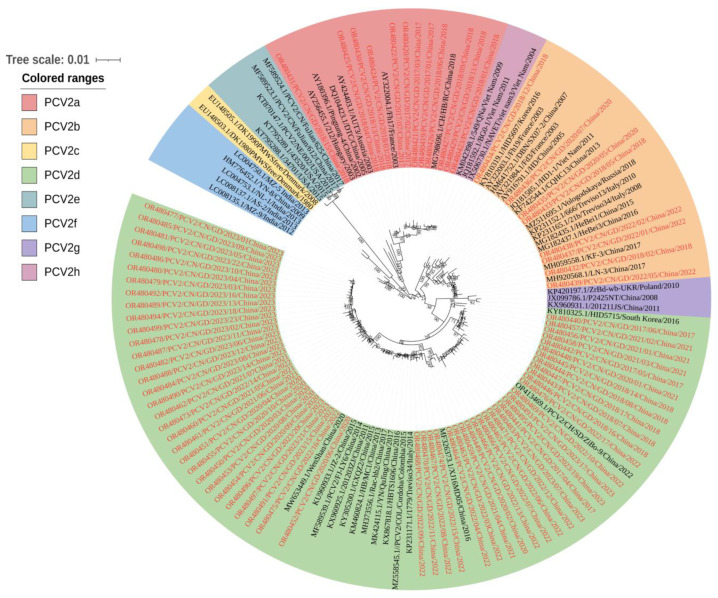
Phylo-genetic analysis of the 80 obtained strains and 52 reference strains based on the complete sequences of PCV2. The phylo-genetic tree was constructed using the Clustal W alignment algorithm of MEGA7.0 with the ML method based on 1000 bootstrap replicates. The different genotypes are represented by different colors and the sequences of this study are indicated by red font in this figure.

**Figure 3:**
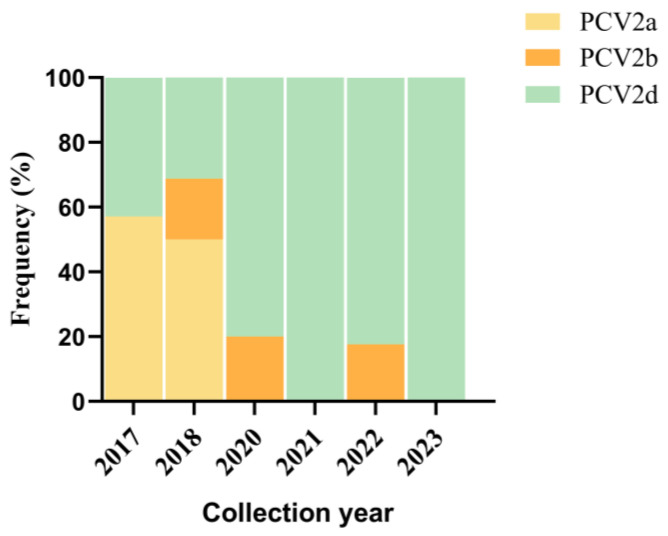
Frequency changes in PCV2 sub-types across years.

A genetic analysis was conducted on the 80 PCV2 complete genomic sequences acquired from the 109 PCV2-positive samples. These sequences were subjected to a phylo-genetic analysis with 52 reference strains reported in the GenBank database (Table S3). The results revealed that PCV2a, PCV2b, and PCV2d accounted for 14.63% (12/80), 9.76% (8/80), and 73.17% (60/80) of the total isolates, respectively (Figure 2). Notably, the prevalence of PCV2 sub-types in Guangdong province transitioned from PCV2a dominance between 2017 and 2018 to PCV2d dominance after 2020, as depicted in Figure 3. A sequence analysis indicated that the 80 strains shared 93.6% to 100% identity in their whole genomes, 96.1% to 100% identity in the ORF1 gene, and 87.0% to 100% identity in the ORF2 gene. In comparison to the 52 referenced PCV2 strains (Table S3), the 132 strains shared 90.1% to 99.9% whole-genome identity, 96.0% to 100% ORF1 gene identity, and 80.0% to 99.4% ORF2 gene identity. Nucleotide mutations in the 80 PCV2 sequences led to the translation of 53 unique Cap proteins and 48 unique Rep proteins. At the amino acid level, the 53 unique Cap proteins shared 84.2% to 99.6% identity with each other, while exhibiting a relatively low identity (79.5% to 100%) to the 52 PCV2 reference strains. Similarly, the 48 Rep proteins displayed a high identity (96.5% to 99.7%) among themselves and with the 52 PCV2 reference strains.

**Figure 4:**
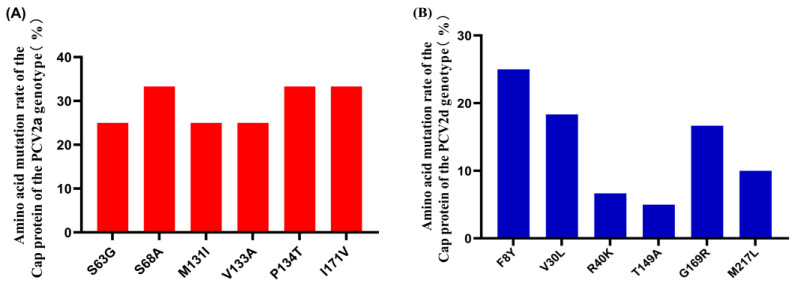
Amino acid mutation ratio in Cap protein of PCV2 isolates. (**A**) The high-frequency mutation ratio of the Cap protein of the PCV2a genotype. (**B**) The high-frequency mutation ratio of the Cap protein of the PCV2d genotype.

The alignment of the Cap protein was performed among the 80 PCV2 isolates in our study (Table S2) and the 52 reference PCV2 strains (Table S3). A subsequent investigation revealed a total of 89 amino acid mutations across the 80 PCV2 isolates (Table S5). Previous studies have identified four epitope regions on the Cap protein, namely, A (51–84 aa), B (113–132 aa), C (161–208 aa), and D (228–234 aa) [51]. Within our study, 16 amino acid mutations were observed in epitope A, 11 in epitope B, 17 in epitope C, and 3 in epitope D. Notably, in the PCV2a isolates, high-frequency mutations occurred at amino acid positions S63G, S68A, M131I, V133A, P134T, and I171V (Figure 4A). Similarly, PCV2d isolates displayed high-frequency mutations at amino acid positions F8Y, V30L, R40K, T149A, G169R, and M217L (Figure 4B). Meanwhile, five high-frequency mutation sites (S63G, S68A, M131I, I171V, and G169R) were localized within the predicted epitope regions of the Cap protein.

### 3.4. Genetic Analysis of PCV3

**Figure 5:**
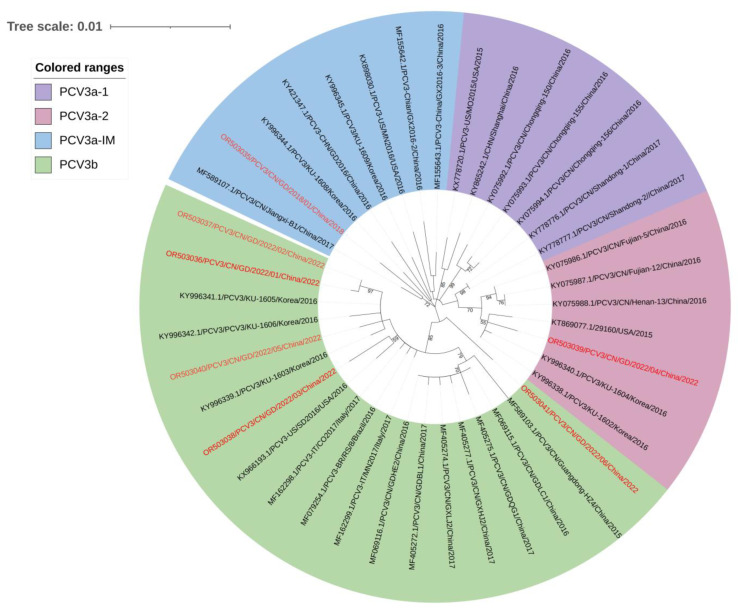
Phylo-genetic trees based on the complete coding sequences (ORF1 + ORF2) of PCV3. The ML tree was constructed with a p-distance model and bootstrapping at 1000 replicates. The PCV3 sub-types were proposed by Li et al. [32]. The different genotypes are represented by different colors and the sequence of this study is indicated by red font, as indicated in the figures.

In our study, seven complete-genome sequences were successfully obtained from the 17 PCV3-positive samples, while only five ORF2 gene sequences were acquired from the remaining 10 positive samples due to poor sample quality. A phylo-genetic tree was constructed based on the combined coding sequences (ORF1 + ORF2) of the 7 PCV3 isolates (Table S2) and 34 reference strains reported in GenBank (Table S4). The analysis revealed that all these PCV3 strains were divided into two major genotypes: PCV3a and PCV3b. Among the seven PCV3 strains in our study, two belonged to PCV3a (specifically PCV3a-IM and PCV3a-2), while the remaining five belonged to PCV3b (Figure 5).

The sequence analysis demonstrated that the genomes of the seven PCV3 isolates were 2000 nt in length. Moreover, these isolates exhibited 96.2% to 100.0% and 99.3% to 99.9% nucleotide similarities in their complete-genome sequences and ORF1, respectively. Among the 12 ORF2 gene sequences obtained from the 17 PCV3-positive samples, nucleotide similarities ranged from 96.9% to 100%. When compared to the 34 reference strains, the seven PCV3 isolates shared 98.7% to 99.7% and 98.8% to 100% nucleotide similarities in their complete-genome sequences and ORF1, respectively. Additionally, the 12 PCV3 ORF2 gene sequences displayed nucleotide similarities ranging from 97.2% to 99.7%. Based on the seven complete genomes and five ORF2 gene sequences of the PCV3-positive samples, the nucleotide mutations resulted in the generation of six unique Rep proteins and eight unique Cap proteins. At the amino acid level, these eight Cap proteins exhibited 96.7% to 99.5% identity with each other, while displaying 96.7% to 100% identity with the 34 reference strains. Similarly, the six unique Rep proteins demonstrated high identity (99.3% to 99.9%) among themselves and with the 34 reference strains. A further investigation uncovered a total of 14 amino acid mutations within the Cap protein. Notably, 12 of these mutations (P11T, H19N, A24V, V48A, G52E, S77T, F104Y, I150L, A152S, T155S, S156G, and F200S) were located within the predicted epitope regions of the PCV3 Cap protein.

## 4. Discussion

PCV2 is widely recognized as the primary causative agent of PCVAD, leading to substantial economic losses in the global swine industry [52,53]. Recent investigations have shown that PCV3 is also prevalent worldwide [22,23,24,25,26,27,28,29,30,31,48]. In this particular study, a comprehensive survey was conducted in Guangdong province, China, spanning from 2017 to 2023, encompassing a total of 193 clinical samples collected from 83 distinct pig farms. These samples were subjected to a conventional PCR analysis to ascertain the prevalence of PCV2, PCV3, and PCV4. The positivity rate for PCV2 was found to be 56.48%, notably higher than previously reported rates in European countries (21%), the Midwestern United States (16.4%), as well as specific regions of China, such as Sichuan province (26.46%) and Jiangxi province (22.5%) [20,54,55,56]. However, the positive rates of PCV2 in the Jilin (86.09%), Henan (62.4%), and Yunnan provinces (60.93%) were higher than those observed in this study [57,58,59]. These discrepancies in data may be attributed to various factors, including geographical location, rearing conditions, sample types, health status, immune responses, sample sizes, PCR methodologies, and overall prevalence, among other variables. Meanwhile, the positive rate for PCV3 was determined to be 8.81%, which was lower compared to rates reported in the Midwestern United States (28.4%) and specific regions of China, such as Yunnan province (49.3%), Guangxi province (37.96%), Shanxi province (80%), and Jiangsu province (14.7%) [22,60,61,62]. These findings suggested a higher prevalence of PCV2 compared to PCV3 in pig herds within Guangdong province. Notably, in 2019, a distinctive PCV genotype, PCV4, was initially identified in nasal swabs and serum samples collected from pigs in Hunan province, China [12]. Subsequent investigations further detected PCV4 in various pig tissue samples (including livers, spleens, kidneys, lungs, lymph nodes, and small intestines) or serum samples from the Shanxi, Henan, Jiangsu, and Guangxi provinces in China [7,8,63,64], albeit with a lower positivity rate compared to PCV2 and PCV3 [7,9,10,63,65]. Furthermore, Hou et al. conducted retrospective studies indicating that PCV4 had been circulating in China for a decade before its initial report [66]. However, in our surveillance efforts conducted in Guangdong province from 2017 to 2023, all clinical specimens (including cerebral tissue, pulmonary tissue, lymph nodes, intestinal tissue, splenic tissue, hepatic tissue, and serum) tested negative for PCV4. Nevertheless, the continued monitoring of PCV4 in the region remains imperative.

Recent studies have shown that co-infections of PCV2 and PCV3 were frequent [54,56]. Specifically, in the Henan province of China, the co-infection rate of PCV2 and PCV3 was documented to reach 69.74% [36]. Compared with previous recorded data, this study’s co-infection rate (8.81%) was significantly lower than those observed in the central (31.18%) and east (31.03%) regions of China [41,67]. However, it was notably higher than the rates reported in European countries (2.6%) [54]. Currently, PCV2, PCV3, CSFV, PRRSV, PRV, and PEDV constitute the six most significant swine pathogens in China. In light of the enduring co-habitation of these viral agents within porcine populations, the phenomenon of co-infection has emerged as a prevalent and recurrent event. This co-occurrence of viral pathogens not only amplifies their pathogenic potential, but also engenders formidable challenges in the realm of disease mitigation and containment, leading to substantial economic ramifications within the global swine industry. Therefore, it is crucial to monitor the co-infection status of PCV2, PCV3, CSFV, PRRSV, PRV, and PEDV. Prior studies have reported that in the southwest of China and the midwest of the USA, the co-infection rates of PCVs with respiratory pathogens such as PRRSV, SIV, or MHP were higher than that of intestinal pathogens like RV, PEDV, PDCoV, or TGEV [20,56]. However, in our study, the co-infection rates of PCVs with intestinal pathogens, particularly PEDV, were higher than those of respiratory pathogens such as CSFV, PRRSV, or PRV. The discrepancy between our results and those previously reported may have occurred because most of our clinical samples were collected from cases exhibiting symptoms of diarrhea.

Since the initial confirmation of PCV2′s prevalence in Chinese swine populations in 2000, there have been two significant genotype shifts: from PCV2a to PCV2b in approximately 2003 and from PCV2b to PCV2d after 2012 [21]. In more recent years, the PCV2d genotype has predominantly been identified globally [41,44,68,69,70]. However, our study revealed that 13 out of 23 complete PCV2 gene sequences were classified as the PCV2a genotype (52.17%), making it the dominant genotype from 2017 to 2018. These data seemed to contradict previous reports [44,70], possibly due to our limited sample size, which may not have accurately reflected the dominant PCV2 genotype during this timeframe. Furthermore, our findings showed that PCV2d became the dominant genotype after 2020 (91.22%), aligning with prior reports [56].

In the phylo-genetic genotyping analysis, the PCV2 strains displayed significant variances. Among the complete genomic sequences of 80 PCV2 strains, 13 were 1768 nt in length, while the remaining 67 strains were 1767 nt long. For ORF1 of the 80 PCV2 strains, it was 945 nt, ORF2 of 21 strains was 702 nt, while ORF2 of the remaining 59 strains was 705 nt. Earlier studies found that compared to PCV2a and PCV2b, PCV2d has an extra amino acid in the C terminus of the Cap protein, resulting in a length of 705 nt instead of 702 nt [41]. Interestingly, in this study, the PCV2/CN/GD/2017/06 strain, which belonged to PCV2d, did not have an extra amino acid in the C terminus of the Cap protein. In circovirus, the Cap protein is the primary structural protein and a key target of the host immune response. Thus, it is necessary to consider amino acid mutations in the Cap protein. In this study, the mutated number and position of the Cap protein in PCV2 isolates were analyzed through a comparison with PCV2 reference strains (Table S3). A total of 89 amino acid mutations were observed on 80 Cap proteins (Table S5) with 6 high-frequency mutation sites on the PCV2a and PCV2d sub-types, respectively (Figure 4). According to previous reports, there were five high-frequency mutation sites in a predicted epitope region in the Cap protein. PCV2′s relatively high nucleotide substitution rate, comparable to RNA viruses, led to the continuously increased genetic diversity and the emergence of new genotypes [71]. The mutational landscape of these amino acids is highly dynamic, potentially enabling the virus to evade both the host’s innate immune response and vaccine-induced immune protection. Despite the significant impact of such mutations on the pathogenicity and immune evasion of PCV2, the precise mechanisms that underlie these effects remain elusive and warrant further exploration.

Since the identification of PCV3 in the USA, its prevalence has spread to countries including Korea, China, Thailand, Japan, Brazil, Italy, Spain, Russia, and Germany, among others [22,23,24,25,26,27,28,29,30,31,48]. Li et al. used the ML, MCC, and NJ methods to construct a phylo-genetic tree based on the PCV3 complete coding sequence (ORF1 + ORF2), with two separate branches being observed; however, the NJ and ML trees constructed using ORF2 did not show clear clusters, especially in the NJ tree [32]. As a result, we performed a phylo-genetic examination based on the complete PCV3 coding sequences (ORF1 + ORF2), which revealed that two out of seven PCV3 strains belonged to PCV3a-2 and PCV3a-IM, respectively, while the remaining five strains belonged to PCV3b. This suggested that PCV3b may be the prevailing genotype in Guangdong province, China, a finding consistent with previous reports conducted between 2018 and 2020 in central China [41]. The homology analysis of the PCV3 strains exhibited a different rate of less than 4%, regardless of the complete-genome sequence, ORF1, ORF2, Rep, or Cap protein, suggesting that the PCV3 strains had higher genetic stability than that of the PCV2 strains, which was consistent with previous reports [43]. At the amino acid level, our study identified 14 amino acid mutation sites on the PCV3 Cap protein sequences, with 12 mutations falling within the predicted epitopes. This could indicate varying immunogenicities conferred by the Cap protein in this cluster of strains.

Vaccination serves as a crucial tool in mitigating the threat of epidemics. Several investigations have demonstrated the efficacy of commercially available PCV2 vaccines in diminishing the incidence of viral infections [72,73,74,75]. Nevertheless, our research showed that PCV2 can still be detected in some pig farms despite the implementation of a PCV2 vaccine, which was consistent with some current reports [54,56]. Consequently, it becomes imperative to delve deeper into the pathogenesis of PCV2 and explore avenues for refining these vaccines.

## 5. Conclusions

This investigation unveiled the widespread dissemination of PCV2 and PCV3 within Guangdong province, China, while no evidence of PCV4 was detected. Through a phylo-genetic analysis, it was evident that the prevalence of the PCV2 sub-types in Guangdong province transitioned from a predominance of PCV2a during the period between 2017 and 2018 to an emergence of PCV2d dominance after 2020. Furthermore, the prevailing genotype of PCV3 appeared to be PCV3b. These findings greatly enhanced our comprehension of the genetic evolution exhibited through circulating strains of PCV2 and PCV3 in China, thereby providing valuable insights for future investigations into these viral agents. Moreover, the occurrence of co-infections between PCVs and other pathogens was shown to be a recurrent phenomenon that can exacerbate clinical symptoms, potentially resulting in substantial economic losses for livestock farms. In summary, this study presented valuable information regarding the prevalence and evolutionary dynamics of PCV2 and PCV3, which should prove instrumental in the development of effective preventive and control strategies moving forward.

## Figures and Tables

**Table 1:** Primer sequences used in this study.

Primers	Sequence 5′–3′	Product Size (bp)	Purpose	References
PEDV S-F	GGTGTTAAGTTTACGTCCCT	468	PEDV detection	
PEDV S-R	AAGTGGGACATAGCCAATAC			
PRRSV ORF7-F	CCAGCCAGTCAATCARCTGTG	292	PRRSV detection	
PRRSV ORF7-R	GCGAATCAGGCGCACWGTATG			
CSFV-F	GCAGAAGCCCACCTCGAGAT	245	CSFV detection	
CSFV-R	TACACCGGTTCCTCCACTCC			
PRV gE-F	ACGAGCCCCGCTTCCACGCG	316	PRV detection	
PRV gE-R	CACCGGTCGCCGAGCAGCGG			
PCV2-DF	CACGGATATTGTAGTCCTGG	500	PCV2 detection	
PCV2-DR	CCGCACCTTCGGATATACTGTC			
PCV3-DF	GATCCACGGAGGTCTGTAGG	357	PCV3 detection	[50]
PCV3-DR	CACGTACCCTTGCAAGTGTG			
PCV4-DF	GTTTTTCCCTTCCCCCACATAG	391	PCV4 detection	[8]
PCV4-DR	ACAGATGCCAATCAGATCTAGGTAC			
PCV2 C-F	GGGCTGGCTGAACTTTTGAAAGTGAGC	1767	Amplify full-length PCV2 genomes	
PCV2 C-R	CCAGCCCGCGGAAATTTCTGACAAACG			
PCV3 C1-F	CGGAGGGAAAGCCCGAAAC	1561	Amplify full-length PCV3 genomes	[49]
PCV3 C1-R	CGCCTAAACGAATGGGAAACT			
PCV3 C2-F	CCGCATAAGGGTCGTCTTG	1011		[6,48]
PCV3 C2-R	TCTTCTCCGCAACTTCAG			
PCV3-F	TTACTTAGAGAACGGACTTGTAACG	651	PCV3 Cap gene amplification	[42]
PCV3-R	AAATGAGACACAGAGCTATATTCAG			

**Table 2:** PCV2 and PCV3 infections observed on farms and in samples during 2017–2023.

Cities	Number of Positive Farms (% Out of Total Number of Farms)					Number of Positive Samples (% Out of Total Number of Samples)				
	*n*	PCV2 (%)	PCV3 (%)	PCV4 (%)	Co-Infection (%)	*n*	PCV2 (%)	PCV3 (%)	PCV4 (%)	Co-Infection (%)
Shaoguan	3	3 (100.00)	0 (0.00)	0	0 (0.00)	19	17 (89.47)	0 (0.00)	0	0 (0.00)
Foshan	36	27 (75.00)	4 (11.11)	0	4 (11.11)	64	43 (67.19)	9 (14.06)	0	9 (14.06)
Guanzhou	7	5 (71.43)	2 (28.57)	0	2 (28.57)	12	8 (66.67)	2 (16.67)	0	2 (16.67)
Maoming	5	4 (80.00)	0 (0.00)	0	0 (0.00)	13	7 (53.85)	0 (0.00)	0	0 (0.00)
Jiangmen	11	7 (63.64)	2 (18.18)	0	2 (18.18)	30	12 (40.00)	2 (6.67)	0	2 (6.67)
Yangjiang	6	1 (16.67)	0 (0.00)	0	0 (0.00)	21	5 (23.81)	0 (0.00)	0	0 (0.00)
Dongguan	5	3 (60.00)	1 (20.00)	0	1 (20.00)	9	6 (66.67)	1 (11.11)	0	1 (11.11)
Qingyuan	6	4 (66.67)	1 (16.67)	0	1 (16.67)	21	9 (42.86)	3 (14.29)	0	3 (14.29)
Zhongshan	4	2 (50.00)	0 (0.00)	0	0 (0.00)	4	2 (50.00)	0 (0.00)	0	0 (0.00)
Total	83	56 (67.47)	10 (12.05)	0	10 (12.05)	193	109 (56.48)	17 (8.81)	0	17 (8.81)

**Table 3:** Co-infection rate of the 109 PCV2-positive samples with other viruses.

Co-Infection Status	Co-Infection Status in 109 PCV2-Positive Samples			
	Virus	Positive Number	Percentage	Total Percentage
Dual infections	PCV3	10	9.17%	38.53%
	PEDV	15	13.76%	
	PRRSV	8	7.34%	
	PRV	9	8.26%	
Triple infections	PCV3 + PEDV	1	0.92%	14.68%
	PRRSV + CSFV	1	0.92%	
	PEDV + PRRSV	5	4.59%	
	PEDV + PRV	4	3.67%	
	PCV3 + PRRSV	4	3.67%	
	PCV3 + PRV	1	0.92%	
Quadruple infections	PCV3 + PEDV + PRRSV	1	0.92%	1.84%
	PEDV + PRRSV + PRV	1	0.92%	
Total	PCV3	17	15.60%	55.05% ^a^
	PEDV	26	23.85%	
	PRRSV	20	18.35%	
	CSFV	1	0.92%	
	PRV	10	9.17%	

^a^ The results showed that 60 of 109 (55.05%) PCV2-positive samples were co-infected with at least one virus.

## Data Availability

All data from this experiment are available from the corresponding author upon request.

## Supplementary Materials

The following supporting information can be downloaded at: https://www.mdpi.com/article/10.3390/ani13233640/s1, Table S1: Positive rates of PCV3 and PCV2 in different types of swine samples during 2017–2023; Table S2: The information of PCV2 and PCV3 strains obtained in this study; Table S3: The information of 52 referenced PCV2 strains; Table S4: The information of 34 referenced PCV3 strains; Table S5: Information about amino acid (AA) mutations in PCV2 ORF2.
