# Supplementary material for: The Prevalence and Genetic Diversity of Porcine Circoviruses (PCVs) during 2017–2023 in Guangdong Province, China
Source: Animals (Basel). 2023 Nov 24;13(23):3640. doi: 10.3390/ani13233640 (PMC10705073; doi:10.3390/ani13233640)
Supplement: Supplementary file 1 [file animals-13-03640-s001.zip › animals-2657267-supplementary.pdf]

Table S1. Positive rates of PCV3 and PCV2 in different types of swine samples during 2017-2023.

Sample Type[a]	PCV3 Positive Rate Positive/Total (%)	PCV2 Positive Rate Positive/Total (%)	Co-infection Rate Positive/Total (%)
Intestine	4/49 (8.16%)	26/49 (53.06%)	4/49(8.16%)
brain	1/8 (12.5%)	6/8 (75.00%)	1/8 (12.50%)
lymph nodes	1/15 (6.67%)	14/15 (93.33%)	1/15 (6.67%)
Lung	2/27 (7.41%)	17/27 (62.96%)	2/27 (7.41%)
Liver	0/2 (0.00%)	1/2 (50%)	0/2 (0.00%)
Spleen	0/2 (0.00%)	1/2 (50%)	0/2 (0.00%)
Serum	1/21 (4.76%)	8/21 (38.10%)	1/21 (4.76%)
T-pool	8/68 (11.76%)	35/68 (46.77%)	8/68 (11.76%)
Unknown	0/1 (0.00%)	1/1 (100%)	0/1 (0.00%)
Total	17/193 (8.81%)	109/193 (56.48%)	17/193 (8.81%)

T-pool: pooled tissue. a: A total of 193 pigs were collected from pigs of different age groups across 83 distinct pig farms in 9 cities within Guangdong province during 2017-2023. Those pigs displayed respiratory symptoms (n=90), neurological symptoms (n=5), sow abortions and stillbirths (n=7), and diarrhea (n=120) were collected for further PCVs detection. Among them, 29 pigs showed both respiratory symptoms and diarrhea.

Table S2. The information of PCV2 and PCV3 strains obtained in this study.

No.	Accession Number	Strains	Country	Year	Host	Length	Genotype
1	OR480419	PCV2/CN/GD/2017/01	China	2017	Domestic pig	1768 bp	PCV2a
2	OR480420	PCV2/CN/GD/2017/02	China	2017	Domestic pig	1768 bp	PCV2a
3	OR480421	PCV2/CN/GD/2017/03	China	2017	Domestic pig	1768 bp	PCV2a
4	OR480441	PCV2/CN/GD/2017/04	China	2017	Domestic pig	1767 bp	PCV2d
5	OR480442	PCV2/CN/GD/2017/05	China	2017	Domestic pig	1767 bp	PCV2d
6	OR480440	PCV2/CN/GD/2017/06	China	2017	Domestic pig	1768 bp	PCV2d
7	OR480422	PCV2/CN/GD/2017/07	China	2017	Domestic pig	1768 bp	PCV2a
8	OR480423	PCV2/CN/GD/2018/01	China	2018	Domestic pig	1768 bp	PCV2a
9	OR480432	PCV2/CN/GD/2018/02	China	2018	Domestic pig	1767 bp	PCV2b
10	OR480424	PCV2/CN/GD/2018/03	China	2018	Domestic pig	1768 bp	PCV2a
11	OR480425	PCV2/CN/GD/2018/04	China	2018	Domestic pig	1768 bp	PCV2a
12	OR480433	PCV2/CN/GD/2018/05	China	2018	Domestic pig	1767 bp	PCV2b
13	OR480426	PCV2/CN/GD/2018/06	China	2018	Domestic pig	1768 bp	PCV2a
14	OR480443	PCV2/CN/GD/2018/07	China	2018	Domestic pig	1767 bp	PCV2d
15	OR480444	PCV2/CN/GD/2018/08	China	2018	Domestic pig	1767 bp	PCV2d
16	OR480427	PCV2/CN/GD/2018/09	China	2018	Domestic pig	1768 bp	PCV2a
17	OR480429	PCV2/CN/GD/2018/11	China	2018	Domestic pig	1768 bp	PCV2a

No.	Accession Number	Strains	Country	Year	Host	Length	Genotype
18	OR480434	PCV2/CN/GD/2018/12	China	2018	Domestic pig	1767 bp	PCV2b
19	OR480430	PCV2/CN/GD/2018/13	China	2018	Domestic pig	1768 bp	PCV2a
20	OR480445	PCV2/CN/GD/2018/14	China	2018	Domestic pig	1767 bp	PCV2d
21	OR480431	PCV2/CN/GD/2018/15	China	2018	Domestic pig	1768 bp	PCV2a
22	OR480446	PCV2/CN/GD/2018/16	China	2018	Domestic pig	1767 bp	PCV2d
23	OR480447	PCV2/CN/GD/2018/17	China	2018	Domestic pig	1767 bp	PCV2d
24	OR480448	PCV2/CN/GD/2020/01	China	2018	Domestic pig	1767 bp	PCV2d
25	OR480449	PCV2/CN/GD/2020/02	China	2020	Domestic pig	1767 bp	PCV2d
26	OR480450	PCV2/CN/GD/2020/03	China	2020	Domestic pig	1767 bp	PCV2d
27	OR480451	PCV2/CN/GD/2020/04	China	2020	Domestic pig	1767 bp	PCV2d
28	OR480435	PCV2/CN/GD/2020/05	China	2020	Domestic pig	1767 bp	PCV2b
29	OR480452	PCV2/CN/GD/2020/06	China	2020	Domestic pig	1767 bp	PCV2d
30	OR480436	PCV2/CN/GD/2020/07	China	2020	Domestic pig	1767 bp	PCV2b
31	OR480453	PCV2/CN/GD/2020/08	China	2020	Domestic pig	1767 bp	PCV2d
32	OR480454	PCV2/CN/GD/2020/09	China	2020	Domestic pig	1767 bp	PCV2d
33	OR480455	PCV2/CN/GD/2020/10	China	2020	Domestic pig	1767 bp	PCV2d
34	OR480456	PCV2/CN/GD/2021/01	China	2021	Domestic pig	1767 bp	PCV2d
35	OR480457	PCV2/CN/GD/2021/02	China	2021	Domestic pig	1767 bp	PCV2d
36	OR480458	PCV2/CN/GD/2021/03	China	2021	Domestic pig	1767 bp	PCV2d
37	OR480459	PCV2/CN/GD/2021/04	China	2021	Domestic pig	1767 bp	PCV2d
38	OR480460	PCV2/CN/GD/2021/05	China	2021	Domestic pig	1767 bp	PCV2d
39	OR480461	PCV2/CN/GD/2021/06	China	2021	Domestic pig	1767 bp	PCV2d
40	OR480462	PCV2/CN/GD/2021/07	China	2021	Domestic pig	1767 bp	PCV2d
41	OR480437	PCV2/CN/GD/2022/01	China	2021	Domestic pig	1767 bp	PCV2b
42	OR480438	PCV2/CN/GD/2022/02	China	2021	Domestic pig	1767 bp	PCV2b
43	OR480463	PCV2/CN/GD/2022/03	China	2021	Domestic pig	1767 bp	PCV2d
44	OR480464	PCV2/CN/GD/2022/04	China	2021	Domestic pig	1767 bp	PCV2d
45	OR480439	PCV2/CN/GD/2022/05	China	2022	Domestic pig	1767 bp	PCV2b
46	OR480465	PCV2/CN/GD/2022/06	China	2022	Domestic pig	1767 bp	PCV2d
47	OR480466	PCV2/CN/GD/2022/07	China	2022	Domestic pig	1767 bp	PCV2d
48	OR480467	PCV2/CN/GD/2022/08	China	2022	Domestic pig	1767 bp	PCV2d
49	OR480468	PCV2/CN/GD/2022/09	China	2022	Domestic pig	1767 bp	PCV2d
50	OR480469	PCV2/CN/GD/2022/10	China	2022	Domestic pig	1767 bp	PCV2d
51	OR480470	PCV2/CN/GD/2022/11	China	2022	Domestic pig	1767 bp	PCV2d
52	OR480471	PCV2/CN/GD/2022/12	China	2022	Domestic pig	1767 bp	PCV2d
53	OR480472	PCV2/CN/GD/2022/13	China	2022	Domestic pig	1767 bp	PCV2d

No.	Accession Number	Strains	Country	Year	Host	Length	Genotype
54	OR480473	PCV2/CN/GD/2022/14	China	2022	Domestic pig	1767 bp	PCV2d
55	OR480474	PCV2/CN/GD/2022/15	China	2022	Domestic pig	1767 bp	PCV2d
56	OR480475	PCV2/CN/GD/2022/16	China	2022	Domestic pig	1767 bp	PCV2d
57	OR480476	PCV2/CN/GD/2022/17	China	2022	Domestic pig	1768 bp	PCV2d
58	OR480477	PCV2/CN/GD/2023/01	China	2023	Domestic pig	1767 bp	PCV2d
59	OR480478	PCV2/CN/GD/2023/02	China	2023	Domestic pig	1767 bp	PCV2d
60	OR480479	PCV2/CN/GD/2023/03	China	2023	Domestic pig	1767 bp	PCV2d
61	OR480480	PCV2/CN/GD/2023/04	China	2023	Domestic pig	1767 bp	PCV2d
62	OR480481	PCV2/CN/GD/2023/05	China	2023	Domestic pig	1767 bp	PCV2d
63	OR480482	PCV2/CN/GD/2023/06	China	2023	Domestic pig	1767 bp	PCV2d
64	OR480483	PCV2/CN/GD/2023/07	China	2023	Domestic pig	1767 bp	PCV2d
65	OR480484	PCV2/CN/GD/2023/08	China	2023	Domestic pig	1767 bp	PCV2d
66	OR480485	PCV2/CN/GD/2023/09	China	2023	Domestic pig	1767 bp	PCV2d
67	OR480486	PCV2/CN/GD/2023/10	China	2023	Domestic pig	1767 bp	PCV2d
68	OR480487	PCV2/CN/GD/2023/11	China	2023	Domestic pig	1767 bp	PCV2d
69	OR480488	PCV2/CN/GD/2023/12	China	2023	Domestic pig	1767 bp	PCV2d
70	OR480489	PCV2/CN/GD/2023/13	China	2023	Domestic pig	1767 bp	PCV2d
71	OR480490	PCV2/CN/GD/2023/14	China	2023	Domestic pig	1767 bp	PCV2d
72	OR480491	PCV2/CN/GD/2023/15	China	2023	Domestic pig	1767 bp	PCV2d
73	OR480492	PCV2/CN/GD/2023/16	China	2023	Domestic pig	1767 bp	PCV2d
74	OR480493	PCV2/CN/GD/2023/17	China	2023	Domestic pig	1767 bp	PCV2d
75	OR480494	PCV2/CN/GD/2023/18	China	2023	Domestic pig	1767 bp	PCV2d
76	OR480495	PCV2/CN/GD/2023/19	China	2023	Domestic pig	1767 bp	PCV2d
77	OR480496	PCV2/CN/GD/2023/20	China	2023	Domestic pig	1767 bp	PCV2d
78	OR480497	PCV2/CN/GD/2023/21	China	2023	Domestic pig	1767 bp	PCV2d
79	OR480498	PCV2/CN/GD/2023/22	China	2023	Domestic pig	1767 bp	PCV2d
80	OR480499	PCV2/CN/GD/2023/23	China	2023	Domestic pig	1767 bp	PCV2d
81	OR503035	PCV3/CN/GD/2018/01	China	2018	Domestic pig	2000 bp	PCV3a-IM
82	OR503042	PCV3/CN/GD/2018/02	China	2018	Domestic pig	645 bp	-
83	OR503043	PCV3/CN/GD/2018/03	China	2018	Domestic pig	645 bp	-
84	OR503036	PCV3/CN/GD/2022/01	China	2022	Domestic pig	2000 bp	PCV3b
85	OR503037	PCV3/CN/GD/2022/02	China	2022	Domestic pig	2000 bp	PCV3b
86	OR503038	PCV3/CN/GD/2022/03	China	2022	Domestic pig	2000 bp	PCV3b
87	OR503039	PCV3/CN/GD/2022/04	China	2022	Domestic pig	2000 bp	PCV3a-2
88	OR503040	PCV3/CN/GD/2022/05	China	2022	Domestic pig	2000 bp	PCV3b
89	OR503041	PCV3/CN/GD/2022/06	China	2022	Domestic pig	2000 bp	PCV3b

No.	Accession Number	Strains	Country	Year	Host	Length	Genotype
90	OR503044	PCV3/CN/GD/2022/07	China	2022	Domestic pig	645 bp	-
91	OR503045	PCV3/CN/GD/2022/08	China	2022	Domestic pig	645 bp	-
92	OR503046	PCV3/CN/GD/2023/01	China	2023	Domestic pig	645 bp	-

Table S3. The information of 52 referenced PCV2 strains.

No.	Accession Number	Strains	Country	Year	Host	Length	Genotype
1	AY146993.1	Pingtung-3	China	2002	-	1768 bp	PCV2a
2	AY180396.1	Pingtung-4	China	2002	-	1768 bp	PCV2a
3	AY256455.1	212	Hungary	2003	Pig	1768 bp	PCV2a
4	AY322004.1	Fh17	France	2003	Pig	1768 bp	PCV2a
5	AY424403.1	AUT3	Austria	2003	Pig	1768 bp	PCV2a
6	DQ104423.1	DTC	China	2005	-	1768 bp	PCV2a
7	MG798696.1	CH/HB/RC	China	2018	Pig	1768 bp	PCV2a
8	AY322003.1	Fh19	France	2003	Pig	1767 bp	PCV2b
9	AY321984.1	Fd3	France	2003	Pig	1767 bp	PCV2b
10	AY916791.1	HD	China	2005	-	1767 bp	PCV2b
11	JQ181585.1	HD1-1	Viet Nam	2011	Pig	1767 bp	PCV2b
12	KF742544.1	CQHC13	China	2013	Pig	1767 bp	PCV2b
13	KP231152.1	666/Treviso13	Italy	2010	Pig	1767 bp	PCV2b
14	KP231165.1	21b/Treviso34	Italy	2008	Pig	1767 bp	PCV2b
15	KY810319.1	HID5697	South Korea	2016	Pig	1768 bp	PCV2b
16	MG182435.1	HeBei1	China	2015	Pig	1767 bp	PCV2b
17	MG182437.1	HeBei3	China	2016	Pig	1767 bp	PCV2b
18	MZ511695.1	Vologodskaya	Russia	2018	Sus scrofa	1767 bp	PCV2b
19	MH920568.1	LN-3	China	2017	Pig	1767 bp	PCV2b
20	MH059558.1	KF-3	China	2017	Pig	1767 bp	PCV2b
21	EU148503.1	DK1980PMWSfree	Denmark	1980	-	1767 bp	PCV2c
22	EU148505.1	DK1990PMWSfree	Denmark	2008	-	1767 bp	PCV2c
23	KM460824.1	HB-MC1	China	2013	Pig	1767 bp	PCV2d
24	KP231171.1	1779/Treviso34	Italy	2014	Pig	1767 bp	PCV2d
25	KU960933.1	JZ-2	China	2015	Pig	1767 bp	PCV2d
26	KX867818.1	HBTS1606	China	2016	Pig	1767 bp	PCV2d
27	KX960925.1	201203ZJ	China	2011	Pig	1767 bp	PCV2d
28	KY305200.1	GXQZ2	China	2015	Pig	1767 bp	PCV2d
29	KY810325.1	HID5715	South Korea	2016	Pig	1767 bp	PCV2d
30	MF326373.1	XJ16MD05	China	2016	Pig	1767 bp	PCV2d

No.	Accession Number	Strains	Country	Year	Host	Length	Genotype
31	MF589539.1	PCV2/FJ-LY6	China	2014	Pig	1767 bp	PCV2d
32	MH373556.1	Rac-hb2	China	2017	Raccoon dog	1767 bp	PCV2d
33	MK424115.1	YN/QuJing	China	2017	Pig	1767 bp	PCV2d
34	MW653449.1	WenShan	China	2020	Pig	1767 bp	PCV2d
35	MZ558545.1	PCV2/COL/Cordoba	Colombia	2015	Sus scrofa	1767 bp	PCV2d
36	OP413469.1	PCV2/CH/SD/ZiBo-9	China	2022	Pig	1767 bp	PCV2d
37	KT795288.1	34701	USA	2015	Sus scrofa	1777 bp	PCV2e
38	KT795289.1	43520	USA	2015	Sus scrofa	1777 bp	PCV2e
39	KT870147.1	PCV2/USA/NE/002	USA	2015	Sus scrofa	1777 bp	PCV2e
40	MF589523.1	PCV2/CN/FuJian/612	China	2017	Pig	1777 bp	PCV2e
41	MF589524.1	PCV2/CN/FuJian/625	China	2017	Pig	1777 bp	PCV2e
42	HM776452.1	YN-8	China	2009	Pig	1767 bp	PCV2f
43	LC004750.1	MZ-5	India	2013	Sus scrofa	1767 bp	PCV2f
44	LC004753.1	NL-1	India	2013	Sus scrofa	1767 bp	PCV2f
45	LC008135.1	MZ-9	India	2012	Sus scrofa	1767 bp	PCV2f
46	LC008137.1	AS-2	India	2013	Sus scrofa	1767 bp	PCV2f
47	JX099786.1	P2425NT	China	2008	Pig	1767 bp	PCV2g
48	KP420197.1	ZrBd-wb-UKR	Poland	2010	Sus scrofa	1767 bp	PCV2g
49	KX960931.1	201211JS	China	2011	Pig	1767 bp	PCV2g
50	KM042398.1	549/QNa	Viet Nam	2009	Pig	1767 bp	PCV2h
51	JQ181592.1	BG0-1	Viet Nam	2011	Pig	1767 bp	PCV2h
52	JX506730.1	NAVET/vietnam3	Viet Nam	2004	Pig	1767 bp	PCV2h

Table S4. The information of 34 referenced PCV3 strains.

No.	Accession Number	Strains	Country	Year	Host	Length	Genotype
1	KY075992.1	PCV3/CN/Chongqing-150	China	2016	Pig	2000 bp	PCV3a-1
2	KY075994.1	PCV3/CN/Chongqing-156	China	2016	Pig	2000 bp	PCV3a-1
3	KY075993.1	PCV3/CN/Chongqing-155	China	2016	Pig	2000 bp	PCV3a-1
4	KY075987.1	PCV3/CN/Fujian-12	China	2016	Pig	2000 bp	PCV3a-2
5	KY996340.1	PCV3/KU-1604	Korea	2016	Pig	2000 bp	PCV3a-2
6	KY996338.1	PCV3/KU-1602	Korea	2016	Pig	2000 bp	PCV3a-2
7	KT869077.1	PCV3/CN/Fujian-5	China	2016	Pig	2000 bp	PCV3a-2
8	KY075986.1	PCV3/CN/Henan-13	China	2016	Pig	2000 bp	PCV3a-2
9	KY075988.1	PCV3/CN/Fujian-12	China	2016	Pig	2000 bp	PCV3a-2
10	KY778777.1	PCV3/CN/Shandong-2	China	2017	Pig	2000 bp	PCV3a-2
11	KY778776.1	PCV3/CN/Shandong-1	China	2017	Pig	2000 bp	PCV3a-2

No.	Accession Number	Strains	Country	Year	Host	Length	Genotype
12	KY865242.1	CHN/Shanghai	China	2016	Pig	2000 bp	PCV3a-IM
13	KX778720.1	PCV3-US/MO2015	USA	2015	Pig	2000 bp	PCV3a-IM
14	MF589107.1	PCV3/CN/Jiangxi-B1	China	2017	Pig	2000 bp	PCV3a-IM
15	KY996344.1	PCV3/KU-1608	Korea	2016	Pig	2000 bp	PCV3a-IM
16	KY996345.1	PCV3/KU-1609	Korea	2016	Pig	2000 bp	PCV3a-IM
17	KY421347.1	PCV3-CHN/GD2016	China	2016	Pig	2000 bp	PCV3a-IM
18	KX898030.1	PCV3-US/MN2016	USA	2016	-	2000 bp	PCV3a-IM
19	MF155643.1	PCV3-China/GX2016-3	China	2016	Pig	2000 bp	PCV3a-IM
20	MF155642.1	PCV3-China/GX2016-2	China	2016	Pig	2000 bp	PCV3a-IM
21	MF405275.1	PCV3/CN/GDQG1	China	2017	Pig	2000 bp	PCV3b
22	MF405274.1	PCV3/CN/GXLJ2	China	2017	Pig	2000 bp	PCV3b
23	MF405277.1	PCV3/CN/GXHJ2	China	2017	Pig	2000 bp	PCV3b
24	MF405272.1	PCV3/CN/GDBL1	China	2017	Pig	2000 bp	PCV3b
25	MF069115.1	PCV3/CN/GDLC1	China	2016	Pig	2000 bp	PCV3b
26	MF069116.1	PCV3/CN/GDHE2	China	2016	Pig	2000 bp	PCV3b
27	KX966193.1	PCV3-US/SD2016	USA	2016	Pig	2000 bp	PCV3b
28	MF589103.1	PCV3/CN/Guangdong-HZ4	China	2015	Pig	2000 bp	PCV3b
29	KY996342.1	PCV3/KU-1606	Korea	2016	Pig	2000 bp	PCV3b
30	MF162298.1	PCV3-IT/CO2017	Italy	2017	Pig	2000 bp	PCV3b
31	MF079254.1	PCV3-BR/RS/8	Brazil	2016	Pig	2000 bp	PCV3b
32	MF162299.1	PCV3-IT/MN2017	Italy	2017	Pig	2000 bp	PCV3b
33	KY996339.1	PCV3/KU-1603	Korea	2016	Pig	2000 bp	PCV3b
34	KY996341.1	PCV3/KU-1605	Korea	2016	Pig	2000 bp	PCV3b

Table S5. Information about amino acid (AA) mutations in PCV2 ORF2.

AA mutant sites	Genotype			Epitopes
	PCV2a (12)	PCV2b(8)	PCV2d(60)	
3	Y(12)	Y(8)	Y(59)/C(1)	
5	R(12)	R(8)	R(59)/G(1)	
6	R(12)	R(8)	R(59)/G(1)	
8	Y(1)/F(11)	Y(8)	F(45)/Y(15)	
9	R(12)	R(8)	R(59)/C(1)	
12	R(12)	R(8)	R(59)/G(1)	
19	L(12)	L(8)	L(59)/P(1)	
26	R(12)	R(8)	R(59)/H(1)	
28	W(12)	W(8)	W(59)/R(1)	
29	L(12)	L(8)	L(59)/I(1)	
30	V(12)	V(7)/A(1)	V(46)/L(11)/I(3)	

AA mutant sites	Genotype			Epitopes
	PCV2a (12)	PCV2b(8)	PCV2d(60)	
37	R(12)	R(8)	R(59)/H(1)	
40	R(12)	R(8)	R(56)/K(4)	
47	A(11)/S(1)	T(8)	T(60)	
53	F(12)	F(8)	I(58)/T(2)	A(51-84aa)
56	T(12)	T(8)	T(59)/S(1)	
57	V(12)	I(8)	V(58)/A(2)	
58	K(11)/E(1)	K(8)	K(59)/V(1)	
59	A(12)	K(8)/R(1)	K(60)	
60	T(11)/S(1)	T(8)	T(58)/A(2)	
62	V(12)	V(8)	V(58)/A(2)	
63	S(8)/G(3)/T(1)	R(7)/K(1)	R(60)	
64	T(11)/A(1)	T(8)	T(60)	
68	S(8)/A(4)	A(8)	N(60)	
69	V(11)/A(1)	V(8)	V(58)/A(2)	
72	L(12)	M(8)	M(60)	
76	L(11)/I(1)	I(8)	I(60)	
77	D(12)	N(8)	N(60)	
80	V(11)/L(1)	V(8)	V(59)/L(1)	
81	P(12)	P(8)	P(59)/L(1)	
86	T(12)	S(8)	S(60)	distinguish between PCV2a and PCV2b
88	K(12)	P(8)	P(60)	
89	I(12)	R(8)	L(60)	
90	S(12)	S(8)	T(60)	
91	I(12)	V(8)	V(59)/A(1)	
97	R(12)	R(8)	R(59)/G(1)	
99	R(12)	R(8)	R(59)/K(1)	
101	V(12)	V(8)	V(59)/A(1)	
115	D(12)	D(7)/G(1)	D(60)	B(113-132aa)
116	R(12)	R(8)	R(58)/G(1)/K(1)	
120	S(12)	S(8)	S(59)/P(1)	
121	S(12)	S(8)	T(60)	B(113-132aa)
123	I(11)/A(1)	V(8)	V(58)/I(2)	
124	I(11)/F(1)	I(8)	I(60)	
126	D(12)	D(8)	D(59)/N(1)	
128	N(12)	N(8)	N(59)/D(1)	
129	F(12)	F(8)	F(59)/L(1)	
130	V(11)/F(1)	V(8)	V(60)	
131	M(8)/I(3)/P(1)	T(8)	T(60)	
133	V(8)/A(3)/S(1)	A(8)	A(60)	
134	P(8)/T(4)	T(8)	N(60)	

AA mutant sites	Genotype			
	PCV2a (12)	PCV2b(8)	PCV2d(60)	
135	A(12)	A(8)	A(59)/V(1)	
136	Q(11)/L(1)	L(8)	L(60)	
144	Y(12)	Y(8)	Y(59)/H(1)	
149	T(12)	T(8)	T(57)/A(3)	
151	P(12)	T(8)	T(60)	
154	F(12)	F(8)	F(59)/S(1)	
156	Y(12)	Y(8)	Y(59)/H(1)	
160	Y(12)	Y(8)	Y(59)/C(1)	
161	F(12)	F(8)	F(59)/L(1)	
163	P(12)	P(8)	P(59)/S(1)	
164	K(12)	K(8)	K(59)/E(1)	
166	V(12)	V(8)	V(59)/A(1)	
167	L(11)/F(1)	L(8)	L(59)/R(1)	
168	D(11)/G(1)	D(8)	D(60)	
169	S(12)	S(8)	G(50)/R(10)	
171	I(8)/V(4)	I(8)	I(60)	
180	R(12)	R(8)	R(59)/K(1)	C(161-207aa)
185	M(12)	L(8)	L(60)	
186	R(11)/K(1)	R(8)	R(60)	
187	L(11)/I(1)	I(8)	I(59)/L(1)	
190	S(12)	T(7)/A(1)	T(60)	
191	R(11)/K(1)	G(8)	G(60)	
196	V(12)	V(8)	V(59)/A(1)	
199	G(12)	G(8)	G(59)/D(1)	
206	K(12)	I(8)	I(59)/V(1)	
208	D(12)	D(8)	D(59)/G(1)	
210	D(12)	E(8)	D(59)/G(1)	
211	Y(11)/N(1)	Y(8)	Y(58)/H(1)/C(1)	
212	N(12)	N(8)	N(59)/D(1)	
215	V(12)	V(8)	I(59)/V(1)	
216	T(12)	T(8)	T(59)/A(1)	
217	M(12)	M(8)	M(53)/L(6)/I(1)	
218	Y(12)	Y(8)	Y(59)/S(1)	
219	V(12)	V(8)	V(59)/I(1)	
223	E(12)	E(7)/G(1)	E(60)	
230	P(11)/A(1)	P(8)	P(60)	D(228-233aa)
232	K(11)/N(1)	N(8)	N(58)/K(1)/S(1)	
234	K/*	*	K(59)/*(1)	
